# Osteomyelitis: An Uncommon Complication of BCG Vaccination

**DOI:** 10.5704/MOJ.2103.026

**Published:** 2021-03

**Authors:** D Pancharatnam, SM Yong, SC Tan

**Affiliations:** 1 Department of Orthopaedic Surgery, University of Malaya, Kuala Lumpur, Malaysia; 2 Department of Orthopaedic Surgery, Tung Shin Hospital, Kuala Lumpur, Malaysia; 3 Department of Paediatric, Tung Shin Hospital, Kuala Lumpur, Malaysia

Dear editor,

Musculoskeletal tuberculosis (TB) in infants is an important challenge that is often neglected or misdiagnosed due to non-specific clinical presentations and its radiographic appearance is similar to pyogenic osteomyelitis^1^. The difficulty and delay in diagnosis is further compounded by the difficulty in assessing pain in infants.

Recently we came across two children under the age of one-year-old that presented with localised swellings of the left knee and right proximal tibia with reduced movement of the affected limb. Both children were active and feeding well, while one patient had pyrexia.

**Fig. 1:**
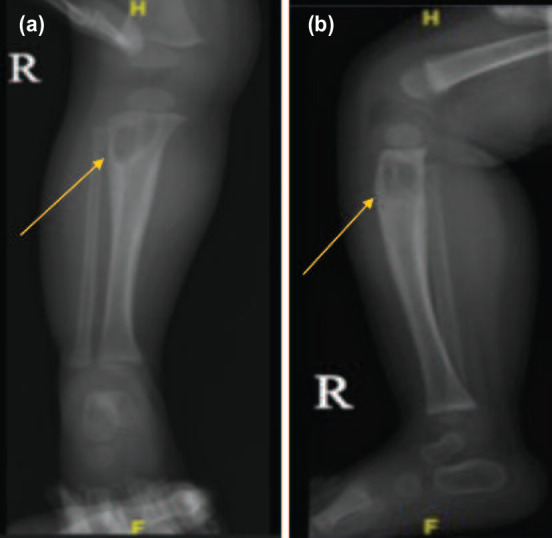
Well-defined radiolucent bone lesions at metaphysis with reactive periosteum thickening seen on (a) right tibia AP view, (b) right tibia lateral view.

**Fig. 2:**
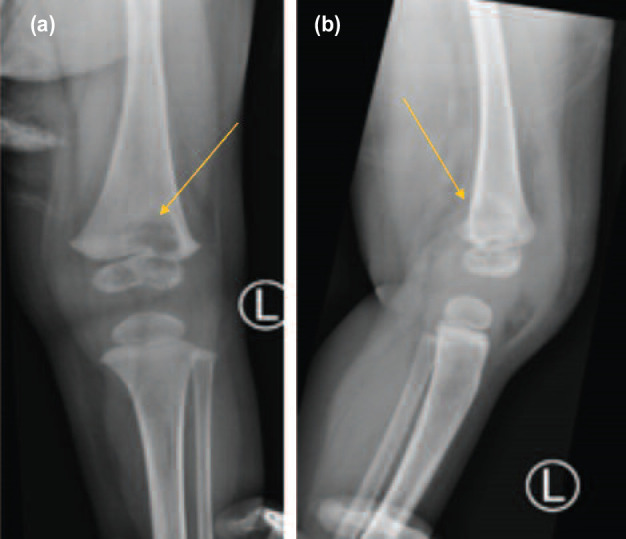
Metaphyseal involvement with extension across the physeal plate with knee swelling seen on (a) left femur AP view, (b) left femur lateral view.

Both patients presented to us only after two weeks of symptoms. None of the family members had a history of pulmonary TB or consumption of raw cow’s milk and both patients had received Bacille Calmette- Guerin (BCG) vaccination at birth. Radiographs revealed well-defined radiolucent metaphyseal bone lesions with reactive periosteal thickening (Fig. 1). One patient had distal femur involvement that extends to physeal plate (Fig. 2).

**Fig. 3:**
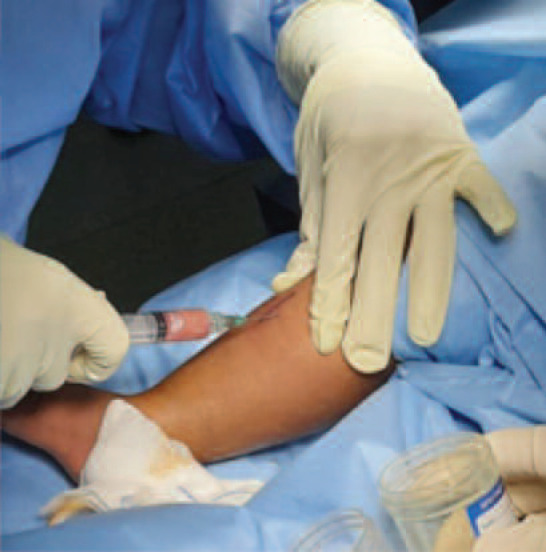
Aspiration of pus from subcutaneous abscess of right proximal tibia region.

Therapeutic and diagnostic surgical intervention was carried out for both patients. Samples were taken from the affected area (Fig. 3) and sent for TB PCR and culture which grew *Mycobacterium bovis*. Histopathology examination showed evidence of chronic necrotising granulomatous inflammation. Oral antituberculous therapy was commenced after establishing the diagnosis and will be completed after 12 months.

The BCG vaccination was first used in 1921 and aims at achieving active immunisation via administration of a live bovine TB strain rendered apathogenic by special culture methods^1,2^. In Malaysia, all neonates are immunised at birth with the BCG vaccine as part of the national childhood immunisation programme to reduce the spread of pulmonary TB and the development of extrapulmonary TB^2^. Koyama *et al* reported the incidence of BCG osteomyelitis to be 0.2 cases per 100000 vaccinations^3^. The interval between BCG vaccination and the development of BCG osteomyelitis is usually five months to five years^2^. The patients were relatively well, had a short duration of pyrexia and their radiographical and histological results were typical of TB^2^. Tissue biopsy is the only method to exclude other diagnoses such as infection and malignancy while the gold standard for checking strain type is by TB PCR testing and culture. Huang *et al* reported that the commonest sites of bone involvement are the extremities especially the lower limb (60%) and the average delay in diagnosis was eight months^4^. Wang *et al* showed excellent results and complete bone healing in their patients who were treated for 12 months with anti TB medications and surgical intervention^1^.

A high index of suspicion of BCG osteomyelitis should be considered in BCG immunised infants who develop localised chronic symptoms such pain and discrete swellings on the extremities. Tissue biopsy, culture and sensitivity, and TB PCR is vital in diagnosis. Early surgical debridement along with appropriate antituberculous therapy has been shown to provide excellent outcome.
